# Supplementary material for: Competing speech streams are simultaneously represented in the human cortex during attention switching
Source: PLoS Biol. 2026 Jul 16;24(7):e3003876. doi: 10.1371/journal.pbio.3003876 (PMC13374923; doi:10.1371/journal.pbio.3003876)
Supplement: Attachment — Submitted filename: ResponseToReviewers_auresp_4.docx [file pbio.3003876.s007.docx]

We thank the Reviewers for their constructive feedback, which has helped us improve the quality of our work.

We have addressed their comments point-by-point below.

**#Reviewer 1**

The authors did a very commendable job with a thoughtful revision process. I found their replies by and large very compelling and have no remaining concerns.
The manuscript will add to a growing interest in the temporal dynamics of cortical states and inherent asymmetries in cortical/cognitive states.

1. As such, the last big paper of the late Peter Lakatos (Nat Neurosci 2016) might be an interesting reference to work into the discussion.

We thank the Reviewer for suggesting this reference, which we found relevant to our work and added to our Discussion section (line 485).

**#Reviewer 2**

The resubmission of the manuscript clarified most of the points and, overall, the revision the reads stronger by including additional statistics and conceptual discussions, congratulations to the authors on the revised manuscript. I have one minor point remaining.

1. The authors clearly responded to R2 minor point 5 (effect of EOG on results), and I agree for the most part. However, as pointed out in the author's reply, this relies on the assumption that EOG is uncorrelated from the speech signal, which in this specific setup might not be guaranteed. In particular frontal channels are affected by EOG and given the simultaneous movement of training and test sets (i.e. performance at timepoint x comes from training and testing around this timepoint), there might be information exploited from EOG. I agree that the effect likely won't have a strong effect on the reported outcomes for engagement/disengagement but especially with the current experimental setup that includes attentional switches between left and right speakers and short analysis windows, I would be more convinced of the results if time lines of performance topographies around the switch and/or performance averages without frontal channels would be provided (in the results,
maybe with [suppl] figure similar to Figure 2A right).

As suggested by the Reviewer, we have examined the scalp topographies of the EEG prediction correlations around the attention switching cue to ensure that the observed effect was not driven by frontal channels, potentially reflecting EOG activity. For both engagement and disengagement processes, the channels more greatly contributing to the model’s prediction appear to be localised in central-parietal areas. The spatial distribution of the topographies appears to be stable across multiple time points, both under sustained attention conditions and closer to the switching cue. We have added a Supplementary figure (S2 Fig, referenced at lines 262-266) showing the time course of EEG prediction correlations for the reference 4-s window, with scalp topographies corresponding to selected time points highlighted across the switching process.
