# Supplementary material for: Competing speech streams are simultaneously represented in the human cortex during attention switching
Source: PLoS Biol. 2026 Jul 16;24(7):e3003876. doi: 10.1371/journal.pbio.3003876 (PMC13374923; doi:10.1371/journal.pbio.3003876)
Supplement: Attachment — Submitted filename: ResponseToReviewers.docx [file pbio.3003876.s005.docx]

We thank the Reviewers for their time in providing constructive feedback about our work. Their considerations have been addressed point-by-point below.

**#Reviewer 1**

1. The first of my more major points is the participant selection for the key temporal-dynamics analyses, where the exclusion of five individuals on the basis of below-chance classification accuracy in either the pre- or post-switch window leaves you with N = 19, which is not unreasonable in absolute terms but, given the idiosyncratic variability one might expect in such a demanding task, calls for a demonstration that the main asymmetry effects hold in the full sample and for some reassurance that the exclusion criterion does not itself bias the temporal profiles toward the reported pattern. This is particularly important because, in studies of task switching in the visual domain—especially where task difficulty or dominance is manipulated—it has been noted that removing extreme-performing participants can influence the magnitude and even the direction of observed asymmetries in engagement and disengagement latencies, though I appreciate the analogy may not map perfectly
here. Nevertheless, regression to the mean and its repercussions (e.g., by removing extreme subjects) is a concern I would appreciate being considered/followed-up here.

We appreciate that this point requires further clarification. Participants were excluded when attention could not be classified above-chance around the switching cue (encoding prediction correlations). In other words, those datapoints were not outliers with respect to our target analysis. An outlier would have been an individual with a start (or end) point of switch substantially different from the others (Figure 2C). Instead, we remove datapoints where those start and end points of switch were meaningless, as auditory attention could not be classified from EEG. Nonetheless, we appreciate the value of verifying that the trends are not affected by the decision of removing those non-decodable participants. To that end, we re-ran the latency analysis without excluding those participants. Our results indicate a similar qualitative pattern as in the previous analysis, with the engagement showing earlier latencies than the disengagement across both the start and end points of the processes. While the trends were consistent with our main results, the introduction of data points where attention could not be classified increases the level of noise of that analysis, weakening the effects below the statistical significance threshold (repeated-measures ANOVA; start point: *F*(1,23) = 2.2, *p* = 0.15, ηp² = 0.03; end point: *F*(1,23) = 2.89, *p* = 0.1, ηp² = 0.04). The revised version of the manuscript acknowledges this result (lines 288-295 in the Results section).

To add some clarity on this potential issue, we investigated whether the temporal patterns of disengagement and engagement were only emerging for participants with high classification accuracy. Specifically, we compared disengagement-engagement latencies with attention classification accuracy as a continuous predictor in a linear mixed-effects (LME) model, with a random intercept for participants. The LME was fit on the difference in latency between disengagement and engagement, by considering all window lengths (1, 2, 4, 8, and 16 seconds), resulting in five data points per participant. This analysis indicated that classification accuracy did not reliably predict latency, neither for the start (*β* = 0.42, *SE* = 3.19, *t*(118) = 0.13, *p* = 0.9, 95% CI [–5.89, 6.73]) nor for the end points (*β* = 5.46, *SE* = 5.99, *t*(118) = 0.91, *p* = 0.36, 95% CI [–6.41, 17.33]) of the processes.

Taken together, these results suggest that the disengagement-engagement temporal asymmetry does not change with classification accuracy in general, and that extremely low (below-chance) classification accuracies add noise to our results, as engagement/disengagement cannot be estimated. We clarified these points in the revised manuscript (please see lines 180-183 and lines 288-295 in the Results section).

**___________________________________________________________________________**

2. Somewhat related to this, I would have liked to see more discussion of whether the observed temporal overlap in neural tracking of both streams is entirely physiological or whether any portion of it could be an artefact of temporal smoothing in the TRF sliding-window procedure; the authors show, to their credit, replications across multiple window lengths, but given the known trade-offs between temporal resolution and stability in these models, it would be valuable to state explicitly how much overlap one might expect from the method alone.

This is a very important point that was also raised by Reviewer 2 (comment 1). The temporal dynamics we measure are impacted by smoothing effects due to the use of encoding windows of different lengths, and do not directly correspond to absolute neural timings. This is an inevitable consequence of calculating correlations over time-windows. For that reason, we have dedicated an entire figure to studying the impact of window-size on the main result of this study (Figure 3). The revised manuscript puts a clearer emphasis on this caveat throughout the manuscript, further expanding on the numerical results in Figure 3 (please also see our reply to Reviewer 2 - comment 1). We found that our result of an engagement-disengagement asymmetry is robust with regard to the choice of the encoding time-window and that, in fact, it only fails when the window is too short (as the attention decoding accuracy becomes less reliable) or too long (with a 16-s window, which is much longer than the switching dynamics).

Another reflection that we added to the manuscript is that a smoothing would similarly impact engagement and disengagement, by stretching them both in time. As such, it could mask but not generate an asymmetry. In other words, if we had instead observed a symmetry of engagement and disengagement, it would have been difficult to determine whether the effect was simply due to the smoothing window masking the effect, or a genuine pattern of our data. We have discussed this point in detail at lines 436-441.

**___________________________________________________________________________**

3. The lexical context-modelling component is perhaps the most intellectually provocative part of the study (although the use of "LLM"s, large language models, could do with a much more detailed methods description.) The result that a Reset model—discarding all prior block context—outperforms richer, switch-aware models is striking, if counterintuitive. Here, I would have expected a more detailed account of why, in your view, the richer models fail to confer a measurable gain: were the semantic regressors for all models standardised to the same scale, could differences in dynamic range contribute to the EEG-prediction differences.

The revised manuscript includes a more in-depth discussion on these points (lines 511-514 and 518-527 in the Discussion section). One consideration we note in the revised manuscript is that our TRF modelling pipeline is insensitive to rescaling of the stimulus features, due to careful normalisation steps and also to our use of correlation metrics for the evaluation (as opposed to regression prediction error). Nonetheless, exploring potential differences in dynamic range across the different lexical expectation models is interesting in itself. First, we compared the average entropy values of the expectation models, finding statistically significant differences (repeated-measures ANOVA, Greenhouse-Geisser correction due to sphericity violation*; F*(1,19) = 39.57, *p* = 9.59e-10, *ηp²* = 0.68). Post-hoc pairwise tests (Holm-adjusted) indicated that the Reset model showed an intermediate average entropy, significantly higher than the Oracle model (*t*(19) = 5.75, *p* = 1.44e-6, Cohen’s *d* = 0.45), and significantly lower than the Attention (*t*(19) = 4.64, *p* = 6.28e-5, Cohen’s *d* = 0.36) and Speaker-Specific (*t*(19) = 2.24, *p* = 0.04, Cohen’s *d* = 0.17) models. As such, the strongest link between Reset model and EEG cannot be attributed to overall larger or weaker entropy values as the values are, in fact, intermediate across the four lexical expectation models tested. These results have been reported at lines 329-338 of the Results section.

Indeed, this is just one of many angles that could be studied for these expectation models. The discussion section in the revised manuscript includes reflections on other possible aspects of these models that should be considered. While we think that it is an interesting result, we acknowledge the need for further validation and inspection across different stimuli, noise conditions, and participants (for example, we are currently investigating this phenomenon with new experiments, one similar to this task but with older adults, the other with young adults after removing the spatial dimension). The other dimension that we discuss is that LLMs like Mistral probably do not deal with switches like the ones in this study as the human brain does. So, there are caveats to be considered when interpreting these results.

Why do you think lexical entropy, but not surprisal, drives the significant context-model effects in this dataset? While I appreciate the authors' caution about overinterpreting the model comparison, the fact that the result converges, at least in broad outline, with findings from certain episodic memory reset paradigms in the working memory literature (which, admittedly, are not speech-specific) could be worth a more mention.

A potential reason is that lexical surprisal is a reactive response, while entropy represent a state of uncertainty that, in principle, should correlate with both anticipatory and reactive neural activity. Surprisal might have been dampened here due to the particular paradigm, where participants are actively switching attention (as opposed to experiments where participants are presented with a single stream that changes abruptly, without a control on what they are attending to). As such, they are “expecting” different speech material because the switch is cued, which may dampen the lexical surprise. In general, it is important to consider that lexical entropy and surprisal are two tightly related regressors, since they both refer to the probability distribution over words in context, with lexical entropy constraining the range of possible surprisal values. For example, low surprisal can result from a context of low entropy, e.g. when a strong expectation is met, or from a context of high entropy, when no clear expectation can be formed. As such, we see the two regressors as complementary and seeing the effect emerging on one of the two is already sufficient evidence, without the need to overextend our interpretation (but we will look further into this with more data in future experiments). We have added a few considerations on the difference between entropy and surprise to the Discussion section (lines 515-523).

**___________________________________________________________________________**

4. Finally, with respect to the alpha ERSP results, the ~4.5 s lag between the encoding switch point and the ERSP minimum appears to be a large and, to my mind, interesting effect. But for a study in the journal, it warrants a deeper look: if alpha decreases here are taken as an index of listening effort, then one has to reconcile the fact that the putative peak of effort reduction occurs well after the neural engagement/disengagement processes appear complete, which is not the pattern one would necessarily predict from classical sustained-attention effort literature. On the other hand, if one views alpha more broadly as a marker of active suppression of irrelevant inputs, then the delayed trough might be understood as the system only gradually releasing suppression on the previous stream—though in that case, the fact that dual-stream tracking is already resolved becomes harder to explain. In short, this is an aspect of the data that could sustain a richer interpretive
discussion but ultimately might require a tighter link to a behavioural marker in order to be usefully interpreted.

The trough of the alpha ERSP time course appears to co-occur with the moment when the new target stream becomes “fully tracked”. Our view is that this may be a “domino effect”, where the newly attended (target) stream becomes easier (i.e., requires less effort) to track, for example due to the availability of sufficient predictive contextual information, both acoustic and linguistic. At the same time, the newly unattended (masker) stream becomes easier to suppress after this tipping point. We appreciate the reviewer’s point on active suppression, and agree that it could be an explanation, but also acknowledge that the ERSP could reflect a combination of different factors.

With regard to the sentence “one has to reconcile the fact that the putative peak of effort reduction occurs well after the neural engagement/disengagement processes appear complete”, we realise that the result in Figure 2C might lead to that reflection. However, it should be noted that the result refers to the tipping point in cortical tracking, where the newly attended stream becomes more strongly tracked than the previously attended stream. In our view, that represents a key point, but not the “completion” of the engagement/disengagement process, which is instead when the separation between cortical tracking of attended and unattended streams returns to pre-switch levels.

Those are valid points, and we have added some reflections to the Discussion (lines 467-478).

4b. Related, I felt that given the obvious and long-time interest in the task- and stimulation-dependent dynamics of alpha oscillatory power and it’s sometimes hard-to-pinpoint relation to cognitive or behavioural outcomes, there might lie a missed opportunity in not analysing the coarse/slow temporal (not so say, causal) relationship between alpha ERSP and the TRF encoding models.

We agree with the Reviewer that it would be interesting to investigate those temporal relationships. In fact, we think that analysis is so valuable that we have been gathering more data (with variations of this task) that, in combination with the present dataset, would provide a more solid basis for drawing interesting conclusions. We think that a dedicated study with cross-dataset validation would be more convincing and valuable than running that analysis here, leaving open questions on whether the result would then replicate. For that reason, and since we had no specific hypotheses on the alpha vs. delta-theta relationships, we prefer to carry out that investigation in a future more targeted study.

**#Reviewer 2**

1.      In the manuscript, the dynamics of switching, for example the 'transient simultaneous encoding of two speech streams' (l.80) is one of the major findings in abstract, results, discussion and conclusion. To be the devil's advocate, I wonder whether it is possible to make such a claim ('transient simultaneous') related to the dynamics given that these results are based on trial averages across windows ≥ 1 second (please correct me if I misunderstood this analysis part). Couldn't such simultaneous tracking be observed because with TRFs we average/accumulate tracking within windows and that the actual following is rapidly fluctuating? Similarly, averaging across trials can also produce such simultaneous/smooth tracking while tracking target and distractor might be sequential or fluctuate. I think this is hard to solve with the TRF approaches as the analysis requires a certain window length. Still, the current results could be seen as an elegant temporal macro view (and I'm impressed the presented results for the 1, 2-second windows that are very short compared to most other tracking studies). If a trial-based estimation of markers (start/end of engagement, disengagement, switching, and also for alpha activity) is possible, this might be a possibility to address part of the point (smoothing/smearing through trial averaging); however, it would certainly push the data.

This is a very important point that was clarified in this revised submission (lines 433-441 in the Discussion). The reviewer noted that measuring a certain period where both streams are tracked (fully or in part) may be influenced by smoothing effects due to the use of a smoothing window and to trial/participant averaging. As such, there is a possibility that the “simultaneous” encoding is, in fact, not simultaneous on the individual trial/participant, but only when looking at these average responses. And we appreciate that looking at that intersection point (i.e., where one would infer the “simultaneous” tracking) is subject to that issue. However, our observation stems from the detection of an engagement-disengagement asymmetry. Neither the smoothing operation nor the trial/participant average could explain this asymmetry (it could stretch it but not explain it). Measuring an earlier engagement-start than the disengagement-start indicates that there is a certain time-frame where both streams are somewhat tracked (albeit not necessarily reflecting a “full” tracking). If our results had, instead, shown an engagement-disengagement symmetry, we agree that detecting high cortical tracking values in the transition window could have been purely due to the smoothing window. However, measuring that asymmetry gives us strong evidence for that simultaneous tracking.

In my opinion, such considerations regarding the interpretation need to be discussed as the attentional switching dynamics are the major point; it should be clear that the current results reflect correlates at the chosen temporal resolution rather than direct evidence of the underlying neural dynamics during switching. This is not meant to undermine the TRF approach that enabled the analysis (and results) in the first place.

The reviewer is right when they say that the results (e.g., the start-end time points for engagement and disengagement) depend on the resolution/smoothing. In other words, measuring the “conclusion” of the engagement process at ~4.5 seconds doesn’t mean that the brain takes 4.5s to do that, as the smoothing window has to be taken into account. Figure 3 reports how those results change with the resolution/smoothing window. We made sure that the revised version highlights this in multiple places (Introduction: lines 78-79; Results: lines 173-177, 255-258; Discussion: lines 435-436; Methods: 674-675).

**___________________________________________________________________________**

2.      Overall, the theoretical connections between the attention switching results (Figs 2, 3) and the semantic analysis (Fig 4) are vague. I might have missed their motivation but in the current manuscript it feels like a separate, exploratory question and it is unclear why other lexical information (e.g. at the phonological level) was not included.

We agree that there are many aspects that could be studied throughout the switch. The revised manuscript clarifies our reasons for focussing on lexical processing (lines 490-503, Discussion), providing a stronger connection between the analyses reported in the manuscript.

The motivation for this analysis is that context is known to play an important role in speech comprehension. We were wondering what happens to the context during an attention switch (where participant actively perform the switch). This analysis required formulating numerical hypotheses, which led to the four alternatives in Figure 4 at the lexical level. The analysis is quite challenging, as the numerical hypotheses generate feature vectors that are quite similar, with remarkable differences only locally around the switch. While a different choice of lexical context (e.g., reset vs. oracle) can lead to differences over a few seconds (especially in the prediction entropy/uncertainty), the time-frame where models would give different feature values would be much shorter for features that are based on units like phonemes, for example, which change at a much faster rate. For this numerical reason, we focussed on a linguistic level with known and widely studied neural signatures, and a long integration window that would give us the best chance of measuring the difference. In summary, we were interested in prediction and the build-up of linguistic context in general, and we considered the lexical level as we considered that the best opportunity for addressing this question.

Another methodological reason is that phonology is highly correlated with the speech acoustics, both at the stimulus level and in the neural responses (Di Liberto et al., 2015; Di Liberto et al., 2019). While separating them is possible, it would be challenging to distinguish different phoneme expectation models over the duration of just a few phonemes after each switch (that would be a quite small amount of data). Similar considerations are valid for other speech properties like prosody (Teoh et al., 2019), where the effect sizes are quite small. TRF-N400s and envelope/acoustic TRFs are quite different in both temporal latencies and scalp topography, making lexical expectations the ideal candidate for studying how predictive context is updated after a switch of auditory attention.

There is another reason that we would like to mention. Lexical predictions may be influenced by context at a time-scale of several seconds. In fact, context defined even a minute ago may be relevant to what will be said next. This makes hypotheses like the “Speaker-specific” model plausible. This possibility could not be studied when considering properties relying more on local context, like prosody or phonology.

In addition, for the provided objective ('to understand how our brains update and use lexical context when switching attention', l. 101) it remains unclear whether the semantic processing (reset) was thought to play a 'causal' role, supporting attentional switching, or is a consequence of attending the new stream, thus co-occurring. If it is one of the former, correlations between N4 amplitude and neural markers from the switching analysis might shed some light and strengthen such notion (also minor point 2). If it is the latter, this might be in line with the finding that a reset model fit the data best and that previous content is not taken into account, possibly due to task demands. This links to the to suggestions in the discussion.

We agree with the reviewer. That would be an interesting relationship to assess. However, the low SNR at the single-participant level makes it challenging to study those correlations (please see details in our answer to minor point 2). Such an analysis would require computing an ERP-style N400 response to the first few words following the switching cue and comparing their latency to the neural tracking metrics of our analysis (intersection point, engagement, disengagement). It will be interesting to conduct a dedicated analysis on this question in a separate study, where we will compare the switching dynamics across younger vs. older populations (a second dataset with a similar paradigm has already been collected). With regards to the interpretation of the Reset model, we mention the possibility that its superiority may reflect a memory reset triggered by the switching cue, in line with episodic memory reset paradigms in the memory literature (lines 511-514).

**___________________________________________________________________________**

3.      Judging from Figure 2, the alpha desynchronization follows well the engagement process. Could it be that alpha suppression is linked to the engagement rather than the switching per se (which is not an independent measure and thus still shows a significant link)? This might also relate to the earlier switching vs the negative alpha peak as the switch will happen earlier compared to the end of the engagement.

This is a very interesting point that we decided to further investigate by computing the correlation between the alpha ERSP trough times and the start and end points of the engagement process. The correlation was computed for each window separately (1s, 2s, 4s, 8 seconds) and, despite variability across window lengths, revealed some statistically significant positive relationships between alpha dip and offset of the engagement (4s window: *Pearson’s r* = 0.55; *p* = 0.015; 8s window: *Pearson’s r* = 0.47; *p* = 0.04). There was however no statistically significant correlation for the remaining windows of 1s (*Pearson’s r* = -0.13; *p* = 0.59) and 2s (*Pearson’s r* = 0.09; *p* = 0.71), probably due to decreased SNR with these short windows in the single-participant estimations. No evidence for a statistically significant correlation between alpha ERSP dip and onset of the engagement process was found across the four window lengths considered (1s-window: *Pearson’s r* = -0.09; *p* = 0.71; 2s-window: *Pearson’s r* = 0.08; *p* = 0.74; 4s-window: *Pearson’s r* = -0.26; *p* = 0.27; 8s-window: *Pearson’s r* = -004; *p* = 0.85). Please note that these results did not undergo multiple-comparison correction. Overall, we think that our pattern of results might indicate a relationship between the alpha dip and the conclusion of the engagement process. However, at present the estimates are too noisy and it is difficult to draw solid conclusions. Future analyses of the current dataset and other datasets that we are collecting using a similar experimental paradigm and different populations (younger vs. older listeners) may offer useful insights into this relationship.

For the sake of completeness, we also correlated the alpha ERSP trough latencies to the temporal dynamics of the disengagement process, finding no significant correlation neither with its onset nor with its offset latencies across the four window lengths.

**Minor points**

1.      Please provide more insight into the behavioural results. As it is an attention switching paradigm, they form the basis for ensuring that participants successfully performed the switches. E.g. you mention that you asked also about the difficulty of switching.

We added reports of the difficulty and the preferential interest (left or right stream) reported by the participants to our *Behavioural* *performance* section. (lines 134-139)

This analysis revealed that the average difficulty of switching was judged to be 3.1 out of 5 on average (SEM 0.11). The preference between left and right streams was overall balanced, with participants choosing the left stream 49.74% of the trials (SEM 1.7%).

**___________________________________________________________________________**

2.      Were the encoding switch time and alpha dip related across participants? If so, this would provide stronger evidence for a functional link between tracking dynamics and neural effort (i.e., TRF measures and alpha activity; l.429). As is, the reported relationship is presented in a largely descriptive way, and the conclusions might be read as implying a stronger association than the analyses support.

We agree that such a result would strengthen the functional link. However, the correlation between encoding switch time and ERSP trough time across participants (for each window separately) was not statistically significant (1s-window: *Pearson’s r* = -0.31; *p* = 0.2; 2s-window: *Pearson’s r* = -0.38; *p* = 0.12; 4s-window: *Pearson’s r* = 0.19; *p* = 0.42; 8s-window: *Pearson’s r* = 0.31; *p* = 0.19). One issue is the SNR, which increases with the smoothing/correlation window and with the cross-participant averaging. As such, non-statistically significant correlations between ERSP and cortical tracking dynamics may be due to the lower SNR on single participant data. Nonetheless, at the group level, there is a robust effect whereby the alpha dip tends to follow the encoding switch, consistent with the proposed functional link between tracking dynamics and neural effort. We have modified the text to clarify this caveat.

**___________________________________________________________________________**

3.      The discussion does not address the null finding for the semantic surprisal but focuses on the positive result for semantic entropy in their interpretation. I suggest discussing these opposing results.

Please see reply to Reviewer 1 - comment 3. We have extended our Discussion section with considerations on the different performance and interpretation of the two semantic regressors (lines 515-523).

**___________________________________________________________________________**

4.      Figure 2: I'm confused about the links between the EEG prediction (2A right), alpha activity (2B right) and encoding switch/alpha min bar plots (2C left). If the plots in the right panels of this figure show averages across participants, shouldn't the bar plots reflect the very same? Here the encoding switch is earlier (~2.5-3s) in the bar plot (2C left) as compared to average results in Fig. 2A (~4s).

We agree with the Reviewer that the apparent discrepancy and our caption are confusing. The encoding switch shown in the line-plot (Fig. 2A) is derived from fitting broken lines to the grand-average tracking curves, whereas the bar plots in Fig. 2C report the mean (± SEM) of encoding switches estimated for the single participant. Averaging time courses before estimating switch points is not equivalent to averaging individual switch times, and this difference creates an apparent discrepancy in the plots. We have clarified in the caption the fact that Fig. 2A (lines 230-232) reflects a broken-line fit on the grand-average neural tracking time courses.

**___________________________________________________________________________**

5.      For the ERSP analysis, ICA was used to clean the data, in particular for eye movement. However, it is not clear whether this also happened for the tracking analysis (l. 564 ff). In particular, for attention switches between left and right speakers, could you indicate whether you think that lateral eye movements might mimic attention shifts in TRFs? If EOG was not corrected for in the TRF analyses, it would be good to briefly indicate why this did not affect results (e.g. in methods, results or discussion). If eye-tracking data was collected, these could be included to show that there was no difference for fixation when attending the right or left stream.

The removal of ocular artifacts with ICA was only applied for the computation of the alpha ERSP, and not for the neural tracking analysis. The reason for this difference lies in the different sensitivity of the two methods to blinks and eye movements. The TRF method estimates a mapping of stimulus to brain response over specific time lags. Ocular artifacts are not time-locked to the fine temporal structure of continuous speech, especially in a multi-talker scenario, and as such they do not systematically covary with the stimulus regressors, which were identified for target and masker separately. A power analysis in the alpha band is instead sensitive to ocular artifacts, whose low-frequency activity can overlap with the measured alpha activity. Since the computation of alpha power is more directly tied to neural dynamics than the TRF method, it is more prone to contamination from these artifacts and as such we decided to remove eye-related components from the signal before computing it. We clarified this point in our Methods section (lines 711-719).

**___________________________________________________________________________**

6.      When using GG correction in rmANOVAs, the dfs were not adjusted. Please provide the adjusted dfs (or the correction factor, epsilon).

Thank you for flagging this. We have fixed the adjusted degrees of freedom and the error terms throughout the manuscript.

**___________________________________________________________________________**

7.      As a suggestion: The manuscript might benefit from linking these results to main attention theories, such as Corbetta and Shulman's framework of dorsal (goal-directed) and ventral (stimulus-driven) attention networks (e.g., Corbetta & Shulman, 2002; Corbetta, Patel & Shulman, 2008). Relating the current findings into such a framework could strengthen the theoretical contribution given ideas on reorienting (e.g. the circuit breaker function, which may be relevant in the top-down switching context by interrupting and supporting disengagement from the previous stream).

We thank the Reviewer for these suggestions which we found interesting and relevant to our work. We added a consideration connecting our findings to that framework in the Discussion (lines 441-445).

**# Reviewer 3 (Lei Wang, signed her report):**

1. Regarding the "lexical prediction mechanisms", why did the authors specifically focus on lexical features rather than other acoustic or semantic features of speech? What is the theoretical justification for considering lexical features as particularly important in the process of attention switching?

We thank the Reviewer for raising this important point. The reason for focusing on lexical features of speech is that the switching task alters the construction of meaning compared to typical sequential semantic processing, making this analysis useful to the characterisation of attention switching. In addition, semantic encoding modulates acoustic and phonological speech encoding, with top-down mechanisms enhancing the acoustic representation of contextually congruent words (Broderick et al., 2019).

In general, speech semantics is usually investigated using regressors related to the predictability of single words, as the brain is known to continuously track the information content of speech (Broderick et al., 2018; Broderick et al., 2022; Heilbron et al., 2022). Nonetheless, there are also models focussed on representing meaning at the sentence-level, which could be useful for investigating the neural representation of “gist of the story”, as mentioned in the Discussion (LCM team, 2024).

Please see our reply to a similar point from Reviewer 2 (major comment 2) for more in-depth considerations on this topic.

**___________________________________________________________________________**

2. The abstract mentions "four numerical hypotheses," yet the introduction does not sufficiently describe them. It would strengthen the paper if the introduction explicitly outlined these hypotheses, including their theoretical basis and connections to previous studies. In the Methods and Results sections, the authors present different attention-switching models (Oracle, Attention, Speaker-Specific, Reset). These models and their underlying assumptions should also be introduced and explained in both the Introduction and Methods sections to ensure conceptual consistency.

We agree with the Reviewer on the need for a more precise explanation of our context models. We have enriched our Discussion section with considerations on the reasons to compare four competing context-accumulation strategies based on semantic regressors (lines 490-503). We have also reformulated our explanation in the Introduction and added theoretical grounds for considering the four context hypotheses (lines 103-111).

**___________________________________________________________________________**

3. Line 79: there is a typographical error, "before before," with an unnecessary repetition.

Thank you for flagging this. It has now been corrected.

**___________________________________________________________________________**

4. Line 88: "and to shift their attention rapidly whenever the arrow changed direction." This phrasing suggests that attention shifts were passively driven by visual cues. How did the authors control for or exclude the potential confounding influence of visual stimulation on attention switching?

While the spatially separated attention switching task may have prompted eye movements during the attention switch, participants were instructed to fixate on a cross at the centre of the screen, and the experiment was conducted in a dark room, to minimise ocular artifacts. In general, ocular movements, even if present, do not systematically covary with the temporal structure of speech content, particularly in a context with competing speakers. As such they are not correlated with the speech regressors used in the TRF analysis, which is in itself robust to artifacts, as it relies on a stimulus-response time-locking and regularisation based on ridge regression. Please see our reply to Reviewer 2 –minor comment 5 for further details. **___________________________________________________________________________**

5. Regarding "a four-alternative forced-choice question," there appears to be inconsistency in the description of the behavioral task. In the Results, the authors report performance on a single-choice question, while in the Methods they describe three such questions. How exactly were the behavioral results derived? The design and scoring of the behavioral task are not entirely clear. For instance, since each trial included multiple attention switches and therefore multiple speech segments, which segment was the focus of the forced-choice question?

We have clarified this point in the Methods section (lines 577-583). (Please also refer to the reply to Reviewer 2 – minor comment 1). Please note that we have also improved the *Behavioural Performance* paragraph in the Results section, reporting the results from all behavioural tasks. As attention alternated between the left and right speech streams over the course of the trial, the question could be about the attended segments of either of the two competing streams.

Can this behavioral task reliably capture participants' attentional state at each attention switch?

The content question was mostly used to encourage participants to stay engaged in the attention switching task. While we would not overextend and consider it a ground truth for attentional state, the behavioural result captures, to an extent, attention to the stimulus, as correct responses required paying attention as instructed by the switch cue. **___________________________________________________________________________**

6. Concerning participant selection ("we selected 19 participants"): the reported behavioral accuracy was above 80% with relatively small variance, yet inclusion was based on performance above 50%. Does this imply substantial inter-subject variability? What were the individual behavioral results? For participants who were excluded, why was their performance significantly worse? Further clarification is needed.

The selection criterion for studying the neural tracking around the switching cue was based on the TRF model’s ability to correctly classify the attended speaker from participants’ EEG activity (not on the behavioural result). The rationale was to exclude from the specific attention switching analyses participants whose neural responses did not accurately reflect the attended speaker, because any estimate of switch-related times would have been meaningless in that case. However, the fact that the model’s classification accuracy was lower than chance around the switch does not imply that those participants were not overall paying attention to the correct stream. Rather, it may indicate that they did not react as rapidly as the remaining participants to the switching cue, lingering on the previously attended stream for a longer time, or that they were distributing their attention more evenly between the two competing streams. Overall, they were able to correctly answer behavioural questions, with scores that were even higher than the average (91.25% correct), suggesting that drawing direct parallels between our behavioural and neural metrics may not be appropriate.

**___________________________________________________________________________**

7. The meaning and rationale of Figure 3 are not entirely clear. How were the "start time" and "end time" defined—solely by the onset and offset of the visual cue? Since actual attention switches may not align exactly with the visual prompts, what does Figure 3 truly compare? Additionally, the distinction and relationship between "window length" and "time" should be further clarified.
Start time and end time are defined based on the average neural tracking time-locked to the switch cue time (EEG prediction correlations). They are not tied to the onset of the switching cue because, as the Reviewer notes, the actual switching process could show different temporal dynamics for different participants. The procedure to identify these time points relies on a broken-line-fit to the single-participant EEG prediction correlation values around the switch for the disengagement pattern (from attended to unattended) and the engagement pattern (from unattended to attended). Since EEG prediction correlations must be correlated using a certain correlation window, Figure 2 shows the result for a selected intermediate window which was expected to offer a sufficiently high SNR, based on the literature. Nonetheless, we report results for all correlation window lengths in Figure 3 (from 1s to 16s considered here). Indeed, start and end points were identified at longer temporal latencies as the window length increased, because the window acts as a smoothing filter. Figure 3 is necessary to clarify that the key result (i.e., the asymmetry between engagement and disengagement processes) is stable across choices of window lengths.

**References**

Broderick, M. P., Anderson, A. J., Di Liberto, G. M., Crosse, M. J., & Lalor, E. C. (2018). Electrophysiological Correlates of Semantic Dissimilarity Reflect the Comprehension of Natural, Narrative Speech. *Curr Biol*, *28*(5), 803-809.e803. <https://doi.org/10.1016/j.cub.2018.01.080>

Broderick, M. P., Anderson, A. J., & Lalor, E. C. (2019). Semantic Context Enhances the Early Auditory Encoding of Natural Speech. *The Journal of Neuroscience*, *39*(38), 7564-7575. <https://doi.org/10.1523/jneurosci.0584-19.2019>

Broderick, M. P., Zuk, N. J., Anderson, A. J., & Lalor, E. C. (2022). More than words: Neurophysiological correlates of semantic dissimilarity depend on comprehension of the speech narrative. *European Journal of Neuroscience*, *56*(8), 5201-5214. <https://doi.org/https://doi.org/10.1111/ejn.15805>

Di Liberto, G. M., O'Sullivan, J. A., & Lalor, E. C. (2015). Low-Frequency Cortical Entrainment to Speech Reflects Phoneme-Level Processing. *Curr Biol*, *25*(19), 2457-2465. <https://doi.org/10.1016/j.cub.2015.08.030>

Di Liberto, G. M., Wong, D., Melnik, G. A., & de Cheveigné, A. (2019). Low-frequency cortical responses to natural speech reflect probabilistic phonotactics. *NeuroImage*, *196*, 237-247. <https://doi.org/10.1016/j.neuroimage.2019.04.037>

Haro, S., Rao, H. M., Quatieri, T. F., & Smalt, C. J. (2022). EEG alpha and pupil diameter reflect endogenous auditory attention switching and listening effort. *European Journal of Neuroscience*, *55*(5), 1262-1277. <https://doi.org/https://doi.org/10.1111/ejn.15616>

Heilbron, M., Armeni, K., Schoffelen, J.-M., Hagoort, P., & de Lange, F. P. (2022). A hierarchy of linguistic predictions during natural language comprehension. *Proceedings of the National Academy of Sciences*, *119*(32), e2201968119. <https://doi.org/doi:10.1073/pnas.2201968119>

LCM team, B., L., Duquenne, P.-A., Elbayad, M., Kozhevnikov, A., Alastruey, B., Andrews, P., Coria, M., Couairon, G., Costa-jussà, M. R., Dale, D., Elsahar, H., Heffernan, K., Janeiro, J. M., Tran, T., Ropers, C., Sánchez, E., San Roman, R., Mourachko, A., Saleem, S., & Schwenk, H. (2024). Large concept models: Language modeling in a sentence representation space. In: arXiv.

Teoh, E. S., Cappelloni, M. S., & Lalor, E. C. (2019). Prosodic pitch processing is represented in delta-band EEG and is dissociable from the cortical tracking of other acoustic and phonetic features. *Eur J Neurosci*, *50*(11), 3831-3842. <https://doi.org/10.1111/ejn.14510>
