# Supplementary material for: Competing speech streams are simultaneously represented in the human cortex during attention switching
Source: PLoS Biol. 2026 Jul 16;24(7):e3003876. doi: 10.1371/journal.pbio.3003876 (PMC13374923; doi:10.1371/journal.pbio.3003876)
Supplement: Peer review history

# Editor report: Decision Letter 0

**DOI:** 10.1371/journal.pbio.3003876.r001

**Authors:** Christian Schnell, PhD

11 Jul 2025

Dear Dr Carta,

Thank you for submitting your manuscript entitled "Simultaneous cortical tracking of competing speech streams during attention switching" for consideration as a Research Article by PLOS Biology.

Your manuscript has now been evaluated by the PLOS Biology editorial staff as well as by an academic editor with relevant expertise and I am writing to let you know that we would like to send your submission out for external peer review.

However, before we can send your manuscript to reviewers, we need you to complete your submission by providing the metadata that is required for full assessment. To this end, please login to Editorial Manager where you will find the paper in the 'Submissions Needing Revisions' folder on your homepage. Please click 'Revise Submission' from the Action Links and complete all additional questions in the submission questionnaire.

Once your full submission is complete, your paper will undergo a series of checks in preparation for peer review. After your manuscript has passed the checks it will be sent out for review. To provide the metadata for your submission, please Login to Editorial Manager (https://www.editorialmanager.com/pbiology) within two working days, i.e. by Jul 13 2025 11:59PM.

If your manuscript has been previously peer-reviewed at another journal, PLOS Biology is willing to work with those reviews in order to avoid re-starting the process. Submission of the previous reviews is entirely optional and our ability to use them effectively will depend on the willingness of the previous journal to confirm the content of the reports and share the reviewer identities. Please note that we reserve the right to invite additional reviewers if we consider that additional/independent reviewers are needed, although we aim to avoid this as far as possible. In our experience, working with previous reviews does save time.

If you would like us to consider previous reviewer reports, please edit your cover letter to let us know and include the name of the journal where the work was previously considered and the manuscript ID it was given. In addition, please upload a response to the reviews as a 'Prior Peer Review' file type, which should include the reports in full and a point-by-point reply detailing how you have or plan to address the reviewers' concerns.

During the process of completing your manuscript submission, you will be invited to opt-in to posting your pre-review manuscript as a bioRxiv preprint. Visit http://journals.plos.org/plosbiology/s/preprints for full details. If you consent to posting your current manuscript as a preprint, please upload a single Preprint PDF.

Feel free to email us at plosbiology@plos.org if you have any queries relating to your submission.

Kind regards,

Christian

Christian Schnell, PhD

Senior Editor

PLOS Biology

cschnell@plos.org

# Peer review: Decision Letter 1

**DOI:** 10.1371/journal.pbio.3003876.r002

**Authors:** Christian Schnell, PhD

1 Sep 2025

Dear Dr Carta,

Thank you for your patience while your manuscript "Simultaneous cortical tracking of competing speech streams during attention switching" was peer-reviewed at PLOS Biology. It has now been evaluated by the PLOS Biology editors, an Academic Editor with relevant expertise, and by several independent reviewers.

In light of the reviews, which you will find at the end of this email, we would like to invite you to revise the work to thoroughly address the reviewers' reports.

As you will see below, the reviewers the reviewers overall like your study and found it interesting. However, they also raised a number concerns that need to be addressed with additional analyses, a better theoretical framing, deeper discussion of the literature, and clarification of the methods.

Given the extent of revision needed, we cannot make a decision about publication until we have seen the revised manuscript and your response to the reviewers' comments. Your revised manuscript is likely to be sent for further evaluation by all or a subset of the reviewers.

In addition to these revisions, you will need to complete some formatting changes, which you will receive in a follow up email. A member of our team will be in touch with a set of requests shortly.

We expect to receive your revised manuscript within 3 months. Please email us (plosbiology@plos.org) if you have any questions or concerns, or would like to request an extension.

At this stage, your manuscript remains formally under active consideration at our journal; please notify us by email if you do not intend to submit a revision so that we may withdraw it.

**IMPORTANT - SUBMITTING YOUR REVISION**

Your revisions should address the specific points made by each reviewer. Please submit the following files along with your revised manuscript:

1. A 'Response to Reviewers' file - this should detail your responses to the editorial requests, present a point-by-point response to all of the reviewers' comments, and indicate the changes made to the manuscript.

*NOTE: In your point-by-point response to the reviewers, please provide the full context of each review. Do not selectively quote paragraphs or sentences to reply to. The entire set of reviewer comments should be present in full and each specific point should be responded to individually, point by point.

You should also cite any additional relevant literature that has been published since the original submission and mention any additional citations in your response.

2. In addition to a clean copy of the manuscript, please also upload a 'track-changes' version of your manuscript that specifies the edits made. This should be uploaded as a "Revised Article with Changes Highlighted" file type.

*Re-submission Checklist*

When you are ready to resubmit your revised manuscript, please refer to this re-submission checklist: https://plos.io/Biology_Checklist

To submit a revised version of your manuscript, please go to https://www.editorialmanager.com/pbiology/ and log in as an Author. Click the link labelled 'Submissions Needing Revision' where you will find your submission record.

Please make sure to read the following important policies and guidelines while preparing your revision:

*Published Peer Review*

Please note while forming your response, if your article is accepted, you may have the opportunity to make the peer review history publicly available. The record will include editor decision letters (with reviews) and your responses to reviewer comments. If eligible, we will contact you to opt in or out. Please see here for more details:

https://blogs.plos.org/plos/2019/05/plos-journals-now-open-for-published-peer-review/

*PLOS Data Policy*

Please note that as a condition of publication PLOS' data policy (http://journals.plos.org/plosbiology/s/data-availability) requires that you make available all data used to draw the conclusions arrived at in your manuscript. If you have not already done so, you must include any data used in your manuscript either in appropriate repositories, within the body of the manuscript, or as supporting information (N.B. this includes any numerical values that were used to generate graphs, histograms etc.). For an example see here: http://www.plosbiology.org/article/info%3Adoi%2F10.1371%2Fjournal.pbio.1001908#s5

*Blot and Gel Data Policy*

We require the original, uncropped and minimally adjusted images supporting all blot and gel results reported in an article's figures or Supporting Information files. We will require these files before a manuscript can be accepted so please prepare them now, if you have not already uploaded them. Please carefully read our guidelines for how to prepare and upload this data: https://journals.plos.org/plosbiology/s/figures#loc-blot-and-gel-reporting-requirements

*Protocols deposition*

To enhance the reproducibility of your results, we recommend that if applicable you deposit your laboratory protocols in protocols.io, where a protocol can be assigned its own identifier (DOI) such that it can be cited independently in the future. Additionally, PLOS ONE offers an option for publishing peer-reviewed Lab Protocol articles, which describe protocols hosted on protocols.io. Read more information on sharing protocols at https://plos.org/protocols?utm_medium=editorial-email&utm_source=authorletters&utm_campaign=protocols

Thank you again for your submission to our journal. We hope that our editorial process has been constructive thus far, and we welcome your feedback at any time. Please don't hesitate to contact us if you have any questions or comments.

Sincerely,

Christian

Christian Schnell, PhD

Senior Editor

PLOS Biology

cschnell@plos.org

------------------------------------

REVIEWS:

Reviewer #1: This manuscript addresses a question that has, until quite recently, been largely left in the shadow of attention paradigms in auditory neuroscience—namely the neural dynamics of switching attention between competing speech streams under naturalistic, multi-talker conditions. This study tackles this with a degree of methodological care and conceptual ambition that is unusual for the field, combining an ecologically valid loudspeaker array and continuous-speech materials with a multi-level analysis that not only separates, with temporal precision, the processes of disengagement from a previous talker and engagement with a new one, but also extends the analysis into the lexical domain by embedding large-language-model-derived predictors in a TRF framework to test competing hypotheses of context updating. I can see why the editors of Plos Biol have taken adequate interest in this.

If proving solid under revision, the finding that engagement reliably precedes disengagement and produces a measurable transient dual-stream cortical representation is both novel and compelling. Also, the observation that alpha-band ERSP minima lag the neural "encoding switch point" by several seconds suggests that attentional reallocation is accompanied by a sustained period of elevated processing demands or inhibitory control. (Here, the functional interpretation is not entirely straightforward and would benefit from explicit engagement with these alternative frameworks, see below.)

In general, there are several points where, in my view, further clarification or elaboration would strengthen the manuscript considerably and should help to bolster the study's main claims. See a list of more major points below.

These observations notwithstanding, I find the study to be a substantial and methodologically forward-looking contribution that will be of real interest not only to auditory and language neuroscientists but also to those in cognitive control, psycholinguistics, and applied domains such as neuro-steered hearing devices, and with the clarifications above—particularly on participant exclusion, possible temporal smearing, and lexical-model interpretation—it should be in a position to make an impact.

1. The first of my more major points is the participant selection for the key temporal-dynamics analyses, where the exclusion of five individuals on the basis of below-chance classification accuracy in either the pre- or post-switch window leaves you with N = 19, which is not unreasonable in absolute terms but, given the idiosyncratic variability one might expect in such a demanding task, calls for a demonstration that the main asymmetry effects hold in the full sample and for some reassurance that the exclusion criterion does not itself bias the temporal profiles toward the reported pattern. This is particularly important because, in studies of task switching in the visual domain—especially where task difficulty or dominance is manipulated—it has been noted that removing extreme-performing participants can influence the magnitude and even the direction of observed asymmetries in engagement and disengagement latencies, though I appreciate the analogy may not map perfectly here. Nevertheless, regression to the mean and its repercussions (e.g., by removing extreme subjects) is a concern I would appreciate being considered/followed-up here.

2. Somewhat related to this, I would have liked to see more discussion of whether the observed temporal overlap in neural tracking of both streams is entirely physiological or whether any portion of it could be an artefact of temporal smoothing in the TRF sliding-window procedure; the authors show, to their credit, replications across multiple window lengths, but given the known trade-offs between temporal resolution and stability in these models, it would be valuable to state explicitly how much overlap one might expect from the method alone.

3. The lexical context-modelling component is perhaps the most intellectually provocative part of the study (although the use of "LLM"s, large language models, could do with a much more detailed methods description.) The result that a Reset model—discarding all prior block context—outperforms richer, switch-aware models is striking, if counterintuitive. Here, I would have expected a more detailed account of why, in your view, the richer models fail to confer a measurable gain: were the semantic regressors for all models standardised to the same scale, could differences in dynamic range contribute to the EEG-prediction differences, and why do you think lexical entropy, but not surprisal, drives the significant context-model effects in this dataset? While I appreciate the authors' caution about overinterpreting the model comparison, the fact that the result converges, at least in broad outline, with findings from certain episodic memory reset paradigms in the working memory literature (which, admittedly, are not speech-specific) could be worth a more mention.

4. Finally, with respect to the alpha ERSP results, the ~4.5 s lag between the encoding switch point and the ERSP minimum appears to be a large and, to my mind, interesting effect. But for a study in the journal, it warrants a deeper look: if alpha decreases here are taken as an index of listening effort, then one has to reconcile the fact that the putative peak of effort reduction occurs well after the neural engagement/disengagement processes appear complete, which is not the pattern one would necessarily predict from classical sustained-attention effort literature. On the other hand, if one views alpha more broadly as a marker of active suppression of irrelevant inputs, then the delayed trough might be understood as the system only gradually releasing suppression on the previous stream—though in that case, the fact that dual-stream tracking is already resolved becomes harder to explain. In short, this is an aspect of the data that could sustain a richer interpertive discussion, but ultimately might require a tighter link to a behavioural marker in order to be usefully interpreted.

4b. Related, I felt that given the obvious and long-time interest in the task- and stimulation-dependent dynamics of alpha oscilltory power and its sometimes hard-to-pinoint relation to cognitive or behavioural outcomes, there might lie a missed opportunity in not analysing the coarse/slow temporal (not so say, causal) relationship between alpha ERSP and the TRF encoding models.

Reviewer #2: Carta and colleagues present an EEG study and results on cortical speech tracking. More specifically, they investigate how attention switches between talkers are processed by the brain using speech tracking metrics of different models as well as posterior alpha activity. Participants were presented with complex auditory scenes including 2 talkers presented via speakers frontally on the right or left side. By means of a visual cue, participants switched their attention either to the right or left speaker during 3-minute trials. Using TRF/tracking approaches and spectral analysis for moving windows, the authors determined the switching dynamics in terms of EEG prediction as well as alpha desynchronization. The main results were early increases of prediction for the (newly) to-be-attended talker as well as later decreases of prediction for the (newly) talker to-be-ignored. In addition, the authors found that alpha suppression was linked descriptively to these tracking measures, in particular the switch between the tracking of the new target and distractor. In a second part, different models (incl predictors for semantic information) were estimated with results showing that a model only taking the current attention section into account could describe EEG responses best.

While the study, experimental design, and results are described thoroughly, I have questions as well as comments that should be addressed before publication.

Main points

1.In the manuscript, the dynamics of switching, for example the 'transient simultaneous encoding of two speech streams' (l.80) is one of the major findings in abstract, results, discussion and conclusion. To be the devil's advocate, I wonder whether it is possible to make such a claim ('transient simultaneous') related to the dynamics given that these results are based on trial averages across windows ≥ 1 second (please correct me if I misunderstood this analysis part). Couldn't such simultaneous tracking be observed because with TRFs we average/accumulate tracking within windows and that the actual following is rapidly fluctuating? Similarly, averaging across trials can also produce such simultaneous/smooth tracking while tracking target and distractor might be sequential or fluctuate. I think this is hard to solve with the TRF approaches as the analysis requires a certain window length. Still, the current results could be seen as an elegant temporal macro view (and I'm impressed with the presented results for the 1, 2-second windows that are very short compared to most other tracking studies). If a trial-based estimation of markers (start/end of engagement, disengagement, switching, and also for alpha activity) is possible, this might be a possibility to address part of the point (smoothing/smearing through trial averaging); however, it would certainly push the data. In my opinion, such considerations regarding the interpretation need to be discussed as the attentional switching dynamics are the major point; it should be clear that the current results reflect correlates at the chosen temporal resolution rather than direct evidence of the underlying neural dynamics during switching. This is not meant to undermine the TRF approach that enabled the analysis (and results) in the first place.

2.Overall, the theoretical connections between the attention switching results (Figs 2, 3) and the semantic analysis (Fig 4) are vague. I might have missed their motivation but in the current manuscript it feels like a separate, exploratory question and it's unclear why other lexical information (e.g. at the phonological level) was not included. In addition, for the provided objective ('to understand how our brains update and use lexical context when switching attention', l. 101) it remains unclear whether the semantic processing (reset) was thought to play a 'causal' role, supporting attentional switching, or is a consequence of attending the new stream, thus co-occurring. If it is one of the former, correlations between N4 amplitude and neural markers from the switching analysis might shed some light and strengthen such notion (also minor point 2). If it is the latter, this might be in line with the finding that a reset model fit the data best and that previous content is not taken into account, possibly due to task demands. This links to the to suggestions in the discussion (l.444 ff).

3.Judging from Figure 2, the alpha desynchronization follows well the engagement process. Could it be that alpha suppression is linked to the engagement rather than the switching per se (which is not an independent measure and thus still shows a significant link)? This might also relate to the earlier switching vs the negative alpha peak as the switch will happen earlier compared to the end of the engagement.

Minor points

1.Please provide more insight into the behavioral results. As it is an attention switching paradigm, they form the basis for ensuring that participants successfully performed the switches. E.g. you mention that you asked also about the difficulty of switching.

2.Were the encoding switch time and alpha dip related across participants? If so, this would provide stronger evidence for a functional link between tracking dynamics and neural effort (i.e., TRF measures and alpha activity; l.429). As is, the reported relationship is presented in a largely descriptive way, and the conclusions might be read as implying a stronger association than the analyses support.

3.The discussion does not address the null finding for the semantic surprisal but focuses on the positive result for semantic entropy in their interpretation. I suggest discussing these opposing results.

4.Figure 2: I'm confused about the links between the EEG prediction (2A right), alpha activity (2B right) and encoding switch/alpha min bar plots (2C left). If the plots in the right panels of this figure show averages across participants, shouldn't the bar plots reflect the very same? Here the encoding switch is earlier (~2.5-3s) in the bar plot (2C left) as compared to average results in Fig. 2A (~4s).

5.For the ERSP analysis, ICA was used to clean the data, in particular for eye movement. However, it is not clear whether this also happened for the tracking analysis (l. 564 ff). In particular, for attention switches between left and right speakers, could you indicate whether you think that lateral eye movements might mimic attention shifts in TRFs? If EOG was not corrected for in the TRF analyses, it would be good to briefly indicate why this did not affect results (e.g. in methods, results or discussion). If eye-tracking data was collected, these could be included to show that there was no difference for fixation when attending the right or left stream.

6.When using GG correction in rmANOVAs, the dfs were not adjusted. Please provide the adjusted dfs (or the correction factor, epsilon).

7.As a suggestion: The manuscript might benefit from linking these results to main attention theories, such as Corbetta and Shulman's framework of dorsal (goal-directed) and ventral (stimulus-driven) attention networks (e.g., Corbetta & Shulman, 2002; Corbetta, Patel & Shulman, 2008). Relating the current findings into such a framework could strengthen the theoretical contribution given ideas on reorienting (e.g. the circuit breaker function, which may be relevant in the top-down switching context by interrupting and supporting disengagement from the previous stream).

Reviewer #3 (Lei Wang, signed her report): 1.Regarding the "lexical prediction mechanisms", why did the authors specifically focus on lexical features rather than other acoustic or semantic features of speech? What is the theoretical justification for considering lexical features as particularly important in the process of attention switching?

2.The abstract mentions "four numerical hypotheses," yet the introduction does not sufficiently describe them. It would strengthen the paper if the introduction explicitly outlined these hypotheses, including their theoretical basis and connections to previous studies. In the Methods and Results sections, the authors present different attention-switching models (Oracle, Attention, Speaker-Specific, Reset). These models and their underlying assumptions should also be introduced and explained in both the Introduction and Methods sections to ensure conceptual consistency.

3.Line 79: there is a typographical error, "before before," with an unnecessary repetition.

4. Line 88: "and to shift their attention rapidly whenever the arrow changed direction." This phrasing suggests that attention shifts were passively driven by visual cues. How did the authors control for or exclude the potential confounding influence of visual stimulation on attention switching?

5.Regarding "a four-alternative forced-choice question," there appears to be inconsistency in the description of the behavioral task. In the Results, the authors report performance on a single-choice question, while in the Methods they describe three such questions. How exactly were the behavioral results derived? The design and scoring of the behavioral task are not entirely clear. For instance, since each trial included multiple attention switches and therefore multiple speech segments, which segment was the focus of the forced-choice question? Can this behavioral task reliably capture participants' attentional state at each attention switch?

6.Concerning participant selection ("we selected 19 participants"): the reported behavioral accuracy was above 80% with relatively small variance, yet inclusion was based on performance above 50%. Does this imply substantial inter-subject variability? What were the individual behavioral results? For participants who were excluded, why was their performance significantly worse? Further clarification is needed.

7.The meaning and rationale of Figure 3 are not entirely clear. How were the "start time" and "end time" defined—solely by the onset and offset of the visual cue? Since actual attention switches may not align exactly with the visual prompts, what does Figure 3 truly compare? Additionally, the distinction and relationship between "window length" and "time" should be further clarified.

# Author comment: Author response to Decision Letter 2

**DOI:** 10.1371/journal.pbio.3003876.r003

12 Mar 2026

Attachment Submitted filename: ResponseToReviewers.docx

# Peer review: Decision Letter 2

**DOI:** 10.1371/journal.pbio.3003876.r004

**Authors:** Christian Schnell, PhD

28 Apr 2026

Dear Dr Carta,

Thank you for your patience while we considered your revised manuscript "Simultaneous cortical tracking of competing speech streams during attention switching" for consideration as a Research Article at PLOS Biology. Your revised study has now been evaluated by the PLOS Biology editors, the Academic Editor and two of the original reviewers.

In light of the reviews, which you will find at the end of this email, we are pleased to offer you the opportunity to address the remaining points from the reviewers in a revision that we anticipate should not take you very long. We will then assess your revised manuscript and your response to the reviewers' comments with our Academic Editor aiming to avoid further rounds of peer-review, although we might need to consult with the reviewers, depending on the nature of the revisions.

In addition to these revisions, you may need to complete some formatting changes, which you will receive in a follow up email. A member of our team will be in touch with a set of requests shortly. If you do not receive a separate email within a few days, please assume that checks have been completed, and no additional changes are required.

We expect to receive your revised manuscript within 1 month. Please email us (plosbiology@plos.org) if you have any questions or concerns, or would like to request an extension.

At this stage, your manuscript remains formally under active consideration at our journal; please notify us by email if you do not intend to submit a revision so that we withdraw the manuscript.

**IMPORTANT - SUBMITTING YOUR REVISION**

Your revisions should address the specific points made by each reviewer. Please submit the following files along with your revised manuscript:

1. A 'Response to Reviewers' file - this should detail your responses to the editorial requests, present a point-by-point response to all of the reviewers' comments, and indicate the changes made to the manuscript.

*NOTE: In your point-by-point response to the reviewers, please provide the full context of each review. Do not selectively quote paragraphs or sentences to reply to. The entire set of reviewer comments should be present in full and each specific point should be responded to individually.

You should also cite any additional relevant literature that has been published since the original submission and mention any additional citations in your response.

2. In addition to a clean copy of the manuscript, please also upload a 'track-changes' version of your manuscript that specifies the edits made. This should be uploaded as a "Revised Article with Changes Highlighted " file type.

*Resubmission Checklist*

When you are ready to resubmit your revised manuscript, please refer to this resubmission checklist: https://plos.io/Biology_Checklist

To submit a revised version of your manuscript, please go to https://www.editorialmanager.com/pbiology/ and log in as an Author. Click the link labelled 'Submissions Needing Revision' where you will find your submission record.

Please make sure to read the following important policies and guidelines while preparing your revision:

*Published Peer Review*

Please note while forming your response, if your article is accepted, you may have the opportunity to make the peer review history publicly available. The record will include editor decision letters (with reviews) and your responses to reviewer comments. If eligible, we will contact you to opt in or out. Please see here for more details:

https://blogs.plos.org/plos/2019/05/plos-journals-now-open-for-published-peer-review/

*PLOS Data Policy*

Please note that as a condition of publication PLOS' data policy (http://journals.plos.org/plosbiology/s/data-availability) requires that you make available all data used to draw the conclusions arrived at in your manuscript. If you have not already done so, you must include any data used in your manuscript either in appropriate repositories, within the body of the manuscript, or as supporting information (N.B. this includes any numerical values that were used to generate graphs, histograms etc.). For an example see here: http://www.plosbiology.org/article/info%3Adoi%2F10.1371%2Fjournal.pbio.1001908#s5

*Blot and Gel Data Policy*

We require the original, uncropped and minimally adjusted images supporting all blot and gel results reported in an article's figures or Supporting Information files. We will require these files before a manuscript can be accepted so please prepare them now, if you have not already uploaded them. Please carefully read our guidelines for how to prepare and upload this data: https://journals.plos.org/plosbiology/s/figures#loc-blot-and-gel-reporting-requirements

*Protocols deposition*

To enhance the reproducibility of your results, we recommend that if applicable you deposit your laboratory protocols in protocols.io, where a protocol can be assigned its own identifier (DOI) such that it can be cited independently in the future. Additionally, PLOS ONE offers an option for publishing peer-reviewed Lab Protocol articles, which describe protocols hosted on protocols.io. Read more information on sharing protocols at https://plos.org/protocols?utm_medium=editorial-email&utm_source=authorletters&utm_campaign=protocols

Thank you again for your submission to our journal. We hope that our editorial process has been constructive thus far, and we welcome your feedback at any time. Please don't hesitate to contact us if you have any questions or comments.

Sincerely,

Christian

Christian Schnell, PhD

Senior Editor

PLOS Biology

cschnell@plos.org

----------------------------------------------------------------

REVIEWS:

Reviewer #1: The authors did a very commendable job with a thoughtful revision process. I found their replies by and large very compelling, and have no remaining concerns.

The manuscript will add to a growing interest in the temporal dynamics of cortical states and inherent asymmetries in cortical/cognitive states. As such, the last big paper of the late Peter Lakatos (Nat Neurosci 2016) might be an interesting reference to work into the discussion.

Reviewer #2: The resubmission of the manuscript clarified most of the points and, overall, the revision the reads stronger by including additional statistics and conceptual discussions, congratulations to the authors on the revised manuscript. I have one minor point remaining.

1.The authors clearly responded to R2 minor point 5 (effect of EOG on results) and I agree for the most part. However, as pointed out in the author's reply, this relies on the assumption that EOG is uncorrelated from the speech signal, which in this specific setup might not be guaranteed. In particular frontal channels are affected by EOG and given the simultaneous movement of training and test sets (i.e. performance at timepoint x comes from training and testing around this timepoint), there might be information exploited from EOG. I agree that the effect likely won't have a strong effect on the reported outcomes fro engagment/disengagement but especially with the current experimental setup that includes attentional switches between left and right speakers and short analysis windows, I would be more convinced of the results if time lines of performance topographies around the switch and/or performance averages without frontal channels would be provided (in the results, maybe with [suppl] figure similar to Figure 2A right).

# Author comment: Author response to Decision Letter 3

**DOI:** 10.1371/journal.pbio.3003876.r005

13 May 2026

Attachment Submitted filename: ResponseToReviewers_auresp_3.docx

# Editor report: Decision Letter 3

**DOI:** 10.1371/journal.pbio.3003876.r006

**Authors:** Christian Schnell, PhD

20 May 2026

Dear Dr Carta,

Thank you for your patience while we considered your revised manuscript "Simultaneous cortical tracking of competing speech streams during attention switching" for publication as a Research Article at PLOS Biology. This revised version of your manuscript has been evaluated by the PLOS Biology editors and the Academic Editor.

Based on our Academic Editor's assessment of your revision, we are likely to accept this manuscript for publication, provided you satisfactorily address the following data and other policy-related requests:

* We would like to suggest a different title as per journal style:

Competing speech streams are simultaneously represented in the human cortex during attention switching

* Please add the links to the funding agencies in the Financial Disclosure statement in the manuscript details.

* Please include information in the Methods section whether the study has been conducted according to the principles expressed in the Declaration of Helsinki.

* DATA POLICY:

You may be aware of the PLOS Data Policy, which requires that all data be made available without restriction: http://journals.plos.org/plosbiology/s/data-availability. For more information, please also see this editorial: http://dx.doi.org/10.1371/journal.pbio.1001797

Note that we do not require all raw data. Rather, we ask that all individual quantitative observations that underlie the data summarized in the figures and results of your paper be made available in one of the following forms:

1) Supplementary files (e.g., excel). Please ensure that all data files are uploaded as 'Supporting Information' and are invariably referred to (in the manuscript, figure legends, and the Description field when uploading your files) using the following format verbatim: S1 Data, S2 Data, etc. Multiple panels of a single or even several figures can be included as multiple sheets in one excel file that is saved using exactly the following convention: S1_Data.xlsx (using an underscore).

2) Deposition in a publicly available repository. Please also provide the accession code or a reviewer link so that we may view your data before publication.

Regardless of the method selected, please ensure that you provide the individual numerical values that underlie the summary data displayed in the following figure panels as they are essential for readers to assess your analysis and to reproduce it: 2C, 4D and S1.

NOTE: the numerical data provided should include all replicates AND the way in which the plotted mean and errors were derived (it should not present only the mean/average values).

Please also ensure that figure legends in your manuscript include information on where the underlying data can be found, and ensure your supplemental data file/s has a legend.

Please ensure that your Data Statement in the submission system accurately describes where your data can be found.

* CODE POLICY

Per journal policy, if you have generated any custom code during the course of this investigation, please make it available without restrictions. Please ensure that the code is sufficiently well documented and reusable, and that your Data Statement in the Editorial Manager submission system accurately describes where your code can be found. More information on our Code Policy, what and how to share can be found here: https://journals.plos.org/plosbiology/s/code-availability

Please note that we cannot accept sole deposition of code in GitHub, as this could be changed after publication. However, you can archive this version of your publicly available GitHub code to Zenodo. Once you do this, it will generate a DOI number, which you will need to provide in the Data Accessibility Statement (you are welcome to also provide the GitHub access information). See the process for doing this here: https://docs.github.com/en/repositories/archiving-a-github-repository/referencing-and-citing-content

As you address these items, please take this last chance to review your reference list to ensure that it is complete and correct. If you have cited papers that have been retracted, please include the rationale for doing so in the manuscript text, or remove these references and replace them with relevant current references. Any changes to the reference list should be mentioned in the cover letter that accompanies your revised manuscript.

In addition to these revisions, you may need to complete some formatting changes, which you will receive in a follow up email. A member of our team will be in touch with a set of requests shortly. If you do not receive a separate email within a few days, please assume that checks have been completed, and no additional changes are required.

We expect to receive your revised manuscript within two weeks.

To submit your revision, please go to https://www.editorialmanager.com/pbiology/ and log in as an Author. Click the link labelled 'Submissions Needing Revision' to find your submission record. Your revised submission must include the following:

- a cover letter that should detail your responses to any editorial requests, if applicable, and whether changes have been made to the reference list

- a Response to Reviewers file that provides a detailed response to the reviewers' comments (if applicable, if not applicable please do not delete your existing 'Response to Reviewers' file.)

- a track-changes file indicating any changes that you have made to the manuscript.

NOTE: If Supporting Information files are included with your article, note that these are not copyedited and will be published as they are submitted. Please ensure that these files are legible and of high quality (at least 300 dpi) in an easily accessible file format. For this reason, please be aware that any references listed in an SI file will not be indexed. For more information, see our Supporting Information guidelines:

https://journals.plos.org/plosbiology/s/supporting-information

*Published Peer Review History*

Please note that you may have the opportunity to make the peer review history publicly available. The record will include editor decision letters (with reviews) and your responses to reviewer comments. If eligible, we will contact you to opt in or out. Please see here for more details:

https://plos.org/published-peer-review-history/

*Press*

Should you, your institution's press office or the journal office choose to press release your paper, please ensure you have opted out of Early Article Posting on the submission form. We ask that you notify us as soon as possible if you or your institution is planning to press release the article.

*Protocols deposition*

To enhance the reproducibility of your results, we recommend that if applicable you deposit your laboratory protocols in protocols.io, where a protocol can be assigned its own identifier (DOI) such that it can be cited independently in the future. Additionally, PLOS ONE offers an option for publishing peer-reviewed Lab Protocol articles, which describe protocols hosted on protocols.io. Read more information on sharing protocols at https://plos.org/protocols?utm_medium=editorial-email&utm_source=authorletters&utm_campaign=protocols

Please do not hesitate to contact me should you have any questions.

Sincerely,

Christian

Christian Schnell, PhD

Senior Editor

cschnell@plos.org

PLOS Biology

# Author comment: Author response to Decision Letter 4

**DOI:** 10.1371/journal.pbio.3003876.r007

9 Jun 2026

Attachment Submitted filename: ResponseToReviewers_auresp_4.docx

# Editor report: Decision Letter 4

**DOI:** 10.1371/journal.pbio.3003876.r008

**Authors:** Christian Schnell, PhD

12 Jun 2026

Dear Sara,

Thank you for the submission of your revised Research Article "Competing speech streams are simultaneously represented in the human cortex during attention switching" for publication in PLOS Biology. On behalf of my colleagues and the Academic Editor, Manuel Malmierca, I am pleased to say that we can in principle accept your manuscript for publication, provided you address any remaining formatting and reporting issues. These will be detailed in an email you should receive within 2-3 business days from our colleagues in the journal operations team; no action is required from you until then. Please note that we will not be able to formally accept your manuscript and schedule it for publication until you have completed any requested changes.

Please take a minute to log into Editorial Manager at http://www.editorialmanager.com/pbiology/, click the "Update My Information" link at the top of the page, and update your user information to ensure an efficient production process.

PRESS

We frequently collaborate with press offices. If your institution or institutions have a press office, please notify them about your upcoming paper at this point, to enable them to help maximize its impact. If the press office is planning to promote your findings, we would be grateful if they could coordinate with biologypress@plos.org. If you have previously opted in to the early version process, we ask that you notify us immediately of any press plans so that we may opt out on your behalf.

We also ask that you take this opportunity to read our Embargo Policy regarding the discussion, promotion and media coverage of work that is yet to be published by PLOS. As your manuscript is not yet published, it is bound by the conditions of our Embargo Policy. Please be aware that this policy is in place both to ensure that any press coverage of your article is fully substantiated and to provide a direct link between such coverage and the published work. For full details of our Embargo Policy, please visit http://www.plos.org/about/media-inquiries/embargo-policy/.

Thank you again for choosing PLOS Biology for publication and supporting Open Access publishing. We look forward to publishing your study.

Sincerely,

Christian

Christian Schnell, PhD

Senior Editor

PLOS Biology

cschnell@plos.org
